# The impact of early-life antibiotics and probiotics on gut microbial ecology and infant health outcomes: a Pregnancy and Birth Cohort in Northwest China (PBCC) study protocol

**DOI:** 10.1186/s12887-022-03811-3

**Published:** 2022-12-28

**Authors:** Qi Qi, Liang Wang, Mitslal Abrha Gebremedhin, Shaoru Li, Xueyao Wang, Jiali Shen, Yingze Zhu, Amanuel Kidane Andegiorgish, Yue Cheng, Lu Shi, Jing Zhou, Ying Yang, Yijun Kang, Wenfang Yang, Zhonghai Zhu, Lingxia Zeng

**Affiliations:** 1 School of Public Health, Xi’an Jiaotong University Health Science Centre, Xi’an, Shaanxi People’s Republic of China; 2 Department of Paediatrics, The Second Affiliated Hospital of Xi’an Jiaotong University, Xi’an, Shaanxi People’s Republic of China; 3 Xi’an Lintong District Maternal and Child Health Hospital, Xi’an, Shaanxi People’s Republic of China; 4 Department of Obstetrics and Gynaecology, The First Affiliated Hospital of Xi’an Jiaotong University, Maternal & Child Health Centre, Xi’an, Shaanxi People’s Republic of China; 5 Key Laboratory of Environment and Genes Related to Diseases (Xi’an Jiaotong University), Ministry of Education, Xi’an, Shaanxi People’s Republic of China

**Keywords:** Cohort, Antibiotics, Probiotics, Microbiota, Maternal and child health

## Abstract

**Background:**

Unreasonable use of antibiotics and probiotics can alter the gut ecology, leading to antibiotic resistance and suboptimal health outcomes during early life. Our study aims are to clarify the association among antibiotic and probiotic exposure in early life, the microecology of the gut microbiota, and the development of antibiotic resistance; to investigate the long-term impact of antibiotics and probiotics on the health outcomes of infants and young children; and to provide a theoretical basis for the rational use of antibiotics and probiotics from a life course perspective.

**Methods:**

The study is a prospective, longitudinal birth cohort study conducted in Shaanxi Province, China from 2018 to 2024. A total of 3,000 eligible mother–child pairs will be enrolled from rural, suburban, and urban areas. The recruitment of the participants begins at pregnancy, and the newborns will be followed up for 2 years at successive timepoints: within 3 days after birth, 42 days after birth, and at 3, 6, 12, 18, and 24 months of age. Sociodemographic data, environmental exposures, dietary patterns, psychological conditions, and medical and drug histories are collected. Cognitive and behavioural development among infants and young children and questionnaires on antibiotic knowledge and behaviour among caregivers will be collected at 12 and 24 months of age. The faecal samples are collected and analysed by 16S rRNA high-throughput sequencing and quantitative PCR (qPCR) for antibiotic resistance genes.

**Discussion:**

The findings will inform antibiotic and probiotic use for pregnant women and infants and contribute to establishing rational use strategies of antibiotics and probiotics for paediatricians, health practitioners, and drug administration policy-makers.

**Trial registration:**

The study was registered on the Chinese Clinical Trial Registry (ChiCTR) platform, http://www.chictr.org.cn (Record ID: ChiCTR2100047531, June 20, 2021).

## Background

The developmental origins of health and disease (DOHaD) concept highlights the importance of the first 1000 days (from conception to one’s second birthday), which is also a critical period for the establishment, gradual diversification, and stability of the gut microbiota. The early life stages are characterized by microbial immobilization and gut microbiota maturation in vivo, and their composition has lifespan implications on subsequent health outcomes [1]. Increasing evidence from epidemiological studies suggests that maturation of the gut microbiota is associated with immune and metabolic development to resist pathogen attack and prevent abnormal immune responses [2, 3]. In this critical phase, the colonization of the gut microbiota is influenced by genetic and environmental factors [4–6], including antibiotic and probiotic use.

Studies have shown that unreasonable antibiotic exposure in early life may have severe consequences for human health, which can impair immune system function, resulting in allergic asthma, gut microbiota disorder, infections, and a vicious cycle of further antibiotic use [7–11]. For maternal and infant health, antibiotic exposure in the prenatal and postnatal periods can disrupt the delicate ecosystem of the newborn microbiome and thus increase the risk of poor health outcomes [6]. Specifically, these health consequences include gut microbiota disorder of an increased microbial abundance up to 3 months after birth [12], a higher risk of obesity among 2-year-olds [13, 14], a higher risk of asthma before 5 years of age [15, 16], and a suboptimal BMI trajectory during early life [17]. However, the dose, timing, and duration of antibiotic exposure and its impact on the health of infants and young children have not been fully studied, especially based on a pregnancy and birth cohort. In underdeveloped districts, misuse and/or overuse of antibiotics during early life is a serious problem in current health practice [18–20], which may result in adverse developmental outcomes and a faster increase in antibiotic resistance. A cross-sectional study in China reported that during 2014–2018, the number of antibiotic prescriptions for pregnant women in western China was 2.1% [21], whereas during recent years, the rate may have increased under COVID-19 restrictions due to self-medication or online buying [22]. In China, antibiotic resistance genes (ARGs) have been detected in newborn faeces [23, 24]. Thus, a well-designed and constructed birth cohort is needed to further clarify the relationship between antibiotics and gut microbiota alterations from an epidemiological perspective.

Probiotics are commonly used in paediatrics and even within families to regulate gut function. Probiotics are commonly defined as “live microorganisms which, when administered in adequate amounts, confer a health benefit to the host” [25]. The Food and Agriculture Organization/World Health Organization (FAO/WHO) consultation indicated that the potential health benefit is dose-based, however, the “adequate amount” in the definition is required to assess human interventions especially in early life periods. Although encouraging, existing conclusions are preliminary without longitudinal verification, and the reasoning behind using probiotics to deal with antibiotic resistance needs more evidence [26, 27].

Improper use of antibiotics and/or probiotics contributes to the development of antibiotic resistance. The strong influence of the infant resistome is mainly through the gut, oral cavity, vagina, and breastmilk, which enrich ARGs [28]. Additionally, some probiotic strains already have natural or mutant antibiotic resistance, and consumption may help restore gut microbiota imbalance caused by antibiotic treatment; however, some specific ARGs can be transmitted vertically, e.g., maternal microbial reservoir transmission during the vaginal delivery period to the infants. Moreover, strains could transfer antibiotic resistance to pathogenic microorganisms and other gastrointestinal commensal microorganisms through horizontal transmission [29]. Combining probiotics with antibiotics can reduce the incidence and severity of antibiotic-associated diarrhoea (AAD), so some special probiotics that do not carry resistance genes are selected to treat or prevent antibiotic side-effects [30]. However, the influence of vertically and horizontally transmitted aberrant microbiota on infants needs more evidence.

To address these issues, the Pregnancy and Birth Cohort in Northwest China (PBCC) was established to (a) clarify the association among antibiotic and probiotic exposure in early life, the microecology of the gut microbiota, and the development of antibiotic resistance; (b) investigate the long-term impact of antibiotics and probiotics on the health outcomes (including birth outcome, physical and cognitive development, disease and immune response) of infants and young children; and (c) provide a theoretical basis for the rational use of antibiotics and probiotics from a life course perspective.

## Methods/design

### Study design

The PBCC is a prospective, ongoing pregnancy and birth cohort study conducted in rural, suburban, and urban areas, which are representative of the different economic levels, geographic coverage, and medical services in Shaanxi Province, Northwest China from January 2018 to December 2024. The rural site is the People’s Hospital in Binzhou city, as well as six town health centres of primary health facilities. The suburban site is Lintong District Maternal and Child Hospital, Xi’an city, which serves a mixed urban and rural population, and the urban site is the Second Affiliated Hospital of Xi’an Jiaotong University. Specifically, the birth numbers at the three sites varied, and the average numbers per year are 1731, 2040, and 3000 at the rural, suburban, and urban sites, respectively. Almost all pregnant women will deliver in hospitals, while some may deliver at home in rural areas (average of 2 per year). The caesarean rate in the three site hospitals varies between 25 and 50%, and the average rate of NICU admission is 10% but varies with hospital level. Critically ill pregnant women will be transferred to higher-level hospitals in advance. After delivery, the exclusive breastfeeding rates declined from 58.3% among newborns to 29.1% at three or four months and declined further to 13.6% among those aged five to six months [31], and the exclusive breastfeeding rates in rural areas, small urban areas and megacities at 6 months were 28.3%, 23.3%, and 35.6%, respectively. After weaning, infants usually receive formula. Child health care centres are usually located in the family’s district within a 1-h walk, and the proportion of children receiving health care is 50%-70%, but this proportion is lower in rural areas because a greater proportion of parents work outside the town.

### Sample size calculation

From the perspective of antibiotic and probiotic use in early life, we considered using probiotics to prevent antibiotic-associated diarrhoea (AAD) in infants to calculate the sample size. The rate of AAD after broad-spectrum antibiotic treatment is reported to be 25%-53% in children [32]. A meta-analysis of probiotic uses to prevent AAD and *Clostridium difficile* infection among hospitalized patients revealed a statistically significant reduction in the risks of AAD (RR = 0.61, 95% CI 0.47 to 0.79) and a lower number of CDI at the end (RR = 0.37, 95% CI 0.22 to 0.61) [33].

Therefore, with a statistical power of 90% (β = 0.1) and a two-tailed significance level of 5% (α = 0.05), for each district, the sample size was calculated using the following formula:

$$n=\frac{{{(z}_{\alpha }\sqrt{2\overline{p }\overline{q}}\pm {z }_{\beta }\sqrt{{p}_{0}{q}_{0}+{p}_{1}{q}_{1}})}^{2}}{{({p}_{1}-{p}_{0})}^{2}}$$

$$p_1=RR\;\cdot\;p_0$$

$$\overline{p }=\frac{1}{2}({p}_{0}+{p}_{1})$$

*where p*_*0*_ denotes the risk of AAD in the control group, *p*_*1*_ denotes the risk of AAD in the treatment group, and $\overline{q }$=1-$\overline{p }$.

Then, we estimated 354 as the minimum number of participants for each group (AAD or no AAD). Considering a 15% foetus attrition rate and 5% loss to follow-up rate, we expanded the number by 20%. Hence, for the three districts, we need to enrol 2,550 participants. Finally, after discussion with the site managers, 3,000 eligible women and their healthy, singleton infants will be recruited for this study.

### Participant enrolment and selection criteria

We collaborate with local doctors, nurses, and medical staff. The pregnant women are enrolled during their first antenatal care (ANC) visits (any trimester) by obstetricians and gynaecologists in the collaborating units. Written informed consent is obtained from the women after enrolment.

The inclusion criteria are as follows: (1) pregnant women who planned to deliver in the collaborating hospital; (2) singleton foetus; and (3) women agreeing to completing at least 2 years of postnatal follow-up. The exclusion criteria are as follows: (1) planning to move out of the town; (2) rejecting or not adhering to the 2-year follow-up; (3) participating in other research projects; and (4) chronic disease or chronic medication use (diagnosed pre-pregnancy). As a result, we will enrol term and preterm infants of any gestation, including neonates who require intensive care, and the medical records will be carefully collected.

### Data collection

**Fig. 1:**
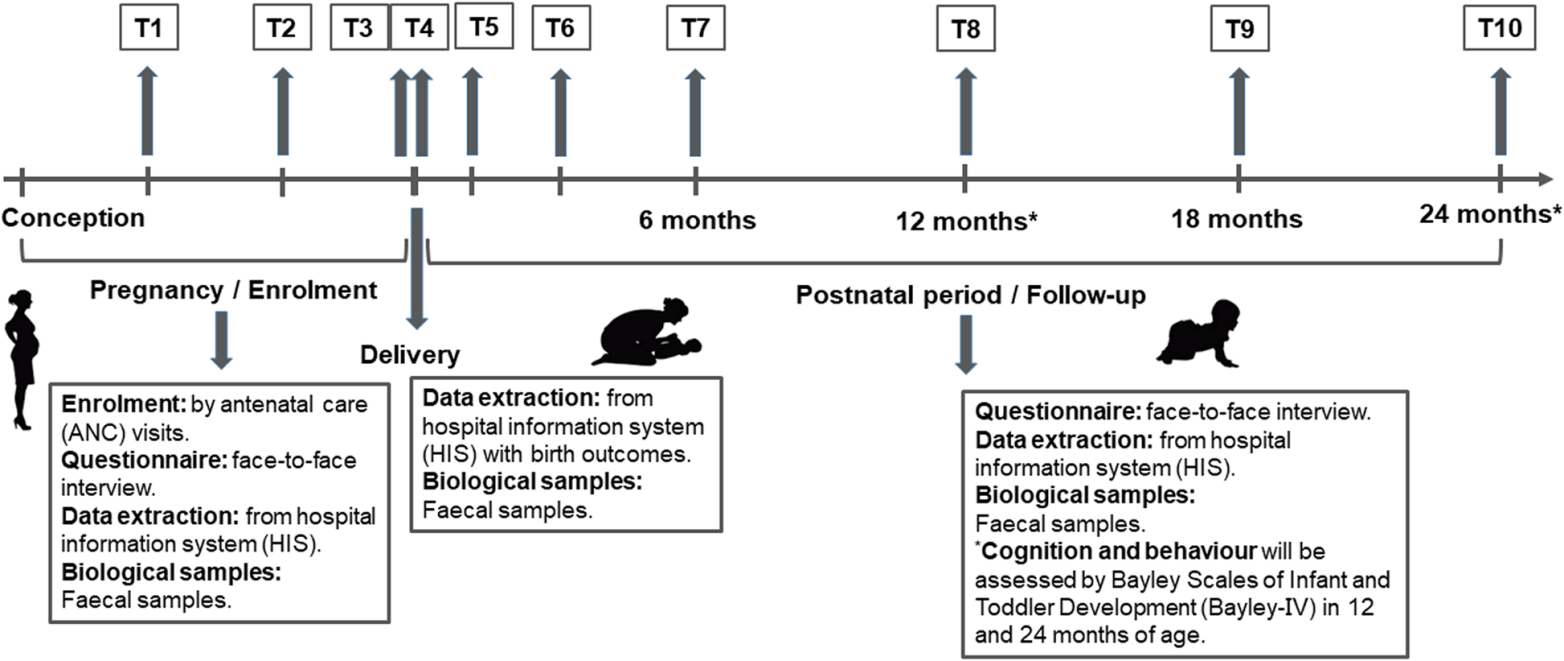
Timeline of the Pregnancy and Birth Cohort in Northwest China (PBCC)

Data collection entails three stages including pregnancy, delivery and after birth, as shown by the timeline in Fig. 1. The ANC, delivery record, and follow-up data from the hospitals are independently managed by the hospital and collected by hospital staff using their routine hospital information system (HIS).

#### Pregnancy (including three trimesters)

During pregnancy (see T1-T3 in Fig. 1), the data will be collected from the ANC visits of the women. Several questionnaires will be carried out face-to-face in the first, second, and third trimesters of pregnancy to assess sociodemographic data, gestational age, environmental exposures, dietary patterns, nutritional supplements, psychological conditions, and medical and drug history, especially antibiotic and probiotic use. After the questionnaire investigation, body composition measurements, including body weight, fat percentage, fat mass, fat-free mass (FFM), muscle mass, total body water (TBW), TBW percentage, bone mass, basal metabolic rate (BMR), visceral fat and body mass index (BMI), will be conducted at the project office by using a body composition analyser (DC-430MA, TANITA Co. Ltd) for pregnant women.

#### Delivery/birth outcomes

After delivery, the delivery record and birth outcomes will be collected by the staff. The meconium will be collected within 3 days (T4 in Fig. 1); most of the meconium samples are the first faeces, and all samples are from the first three bowel movements.

#### After birth (follow-up)

During the afterbirth period, the follow-up time points are 42 days, 3 months, 6 months, 12 months, 18 months, and 24 months of age (T5-T10 in Fig. 1). The follow-up is designed to be embedded in the scheduled immunization and child health services, and therefore the data can be collected regularly in a timely manner. Specifically, the medication and prescription drug data will be obtained by the HIS, and the face-to-face questionnaire is used to collect information on caregivers, feeding patterns, nutritional supplements, and medical and drug history, especially antibiotic and probiotic use.

We also measure the physical development of children at every follow-up time point. We use uniform measurement tools including infant scales, electronic scales, and tape measures. After calibration, weight, height, head circumference, chest circumference, and mid-upper arm circumference are measured at least twice until reaching the minimum standard, and the average is recorded. Furthermore, offspring cognitive and behavioural health will be assessed by Bayley Scales of Infant and Toddler Development (Bayley-IV) at 12 and 24 months of age, which will be conducted by trained research assistants. Likewise, questionnaires on antibiotic knowledge and caregiver behaviours will be collected at these two time points.

### Biological sample collection and analysis

Faecal samples will be collected in three stages (shown in Table 1). The sample collection and laboratory experiments are as follows:

- *Collection and preservation of samples for microbial testing*

We use a one-time sterile collection tube to collect fresh faecal samples with a 75% alcohol-disinfected stencil-sealed cover, which will be transferred quickly within two hours (using an icebox for temporary storage) to the hospital’s clinical laboratory and preserved in a -20 °C refrigerator and frozen after marking. Then, every two weeks, these samples will be temporarily transferred to a mobile fridge, with an average travel time of 60 min, ranging from 30 to 120 min to the lab located at Xi'an Jiaotong University, School of Public Health Centre. All the samples will be stored in a -80 °C refrigerator.

- *DNA extraction and 16S rRNA gene amplicon sequencing*

Bacterial DNA is extracted from faecal samples with a QIAamp Fast DNA Stool Mini Kit (Qiagen, Cat. No./ID: 51,604), and PCR amplification is conducted with barcode-specific bacterial primers targeting the variable regions V3-V4 of the 16S rRNA gene: the forward primer 341F for 5′-CCTAYGGGRBGCASCAG-3’ and 806R for 5′-GGACTACNNGGGTATCTAAT-3’ [34]. Sequencing libraries and paired-end reads are generated on an Illumina HiSeq 2500 platform from Biomarker Technologies Co.Ltd. (Beijing, China) according to a standard protocol.

- *ARGs detected by qPCR*

We then use quantitative PCR (*qPCR*) to detect the expression of ARGs in faecal samples. After a careful literature review, we selected 6 ARGs of four kinds of antibiotics: beta-lactam, tetracycline, fluoroquinolone, and macrolide, which are commonly used during pregnancy and early life [12, 35, 36]. Each sample includes three replicates for data accuracy.

**Table 1:** Faecal sample collection at every timepoint of the Pregnancy and Birth Cohort in Northwest China (PBCC)

Stage	Timepoints	Period	Faecal sample	Sample source
Pregnancy	T1	1^st^ trimester	√	
	T2	2^nd^ trimester	√	Mother
	T3	3^rd^ trimester	√	
Delivery	T4	Birth	√	Infant
After birth	T5	42 days	√	
	T6	3 months	√	
	T7	6 months	√	Infant
	T8	12 months	√	
	T9	18 months	√	
	T10	24 months	√	

### The main study indicators and instructions

#### Independent variables: Antibiotic and probiotic use

To assess antibiotic and probiotic exposure, the information is recorded in detail through the HIS of the hospital including the dose, timing, duration, and type, which are collected not only in the perinatal period but also in the follow-up period. We enquire about maternal or infant administered medications such as antibiotics or probiotics by questionnaire, with the analysis from both simultaneous and separate perspectives to further clarify the effect on the periods of maternal and early life.

#### Outcomes of interest: Birth outcomes and early health outcomes

Birth outcomes include gestational age at delivery (weeks), mode of delivery (vaginal delivery or C-section), gender, infant Apgar scores, infant anthropometric measurements, and medications. The adverse birth outcomes are as follows [37]:

- A low Apgar score is defined as an Apgar score at 5 min of < 7.
- Low birth weight is defined as a live birth weighing < 2500 g.
- Small for gestational age infant (SGA) is defined as birthweight < 10% of that for a specific gestational age.
- Preterm birth is defined as a neonate born before 37 completed weeks.

The secondary health outcomes include antibiotic resistance, disease and treatments, physical development, and cognitive development, and they are recorded at every timepoint.

- Antibiotic resistance: It is reported that antibiotic resistance genes were found in infant faecal samples regardless of antibiotic exposure. In the study, we use 16S rRNA microbiome high-throughput sequencing and qPCR of ARGs for antibiotic resistance gene analysis.
- Disease and treatment: Common diseases in children often occur in the respiratory, digestive, or urinary systems, and the medical treatment and drug history are extracted from the HIS in the hospital.
- Physical development: Anthropometric measurements including weight (kg), height (cm), head circumference (cm), chest circumference (cm), and mid-upper arm circumference (cm), are standardized using WHO norms [37, 38].
- Cognitive development is assessed by standard Bayley Scales of Infant and Toddler Development (Bayley-IV), which is a widely used, comprehensive assessment of development for infants and toddlers aged 1 to 42 months [39] administered by trained research assistants.

### Research management

#### Follow-up compliance

Multiple strategies are used to improve the compliance of the women and infants: (1) Step-by-step training is carried out for the investigators to follow-up the participants. (2) Biological analysis reports are sent to the women regularly so that they can receive health information feedback in time. (3) Social media are used to keep in touch with the women. We use the WeChat application to set up a group to remind the participants of the date of follow-up and sample collection. (4) Calls and home visits are conducted for the participants who are not able to come to the health facilities.

#### Quality control

Regarding the design of the questionnaire, the literature had been extensively reviewed, advice had been obtained from obstetrics and paediatric experts, and preliminary interviews had been conducted before formal enrolment.

Regarding investigator training, everyone will receive a detailed workbook and guidance on how to complete a face-to-face questionnaire and call/home visit at the beginning. During the investigation, disagreements occurring between the two investigators will be resolved by a senior investigator.

After the regular investigation, researchers will check the questionnaire for missing data and logistical errors, and a supplementary survey will be conducted if needed. Data entry will be conducted by hard-copy questionnaire and online investigation, with double-entry assuring data accuracy.

### Data management and statistical analysis

Data will be collected and managed using REDCap (Research Electronic Data Capture) tools by investigators, and questionnaire data entry will be conducted as soon as possible at each timepoint. Moreover, the external data including the records from the HIS, will be inputted regularly. All pregnant women enrolled in the study will be included in the final analysis.

The characteristics of the women or infants are described as the means ± SDs or medians for quantitative variables and percentages with numbers for categorical variables. One-way ANOVA is used to test the difference in means for continuous variables, and chi-square tests or Fisher’s exact test are used to summarize the associations of categorical variables.

To analyse the 16S rRNA gene sequence data, we will not only perform cross-sectional comparisons across individuals at the same time but also perform longitudinal follow-up within the same individual at different time points. All the data will be analysed using QIIME tools. (1) For community diversity and dissimilarity, alpha-diversity metrics including diversity (Shannon, Simpson) and richness (Chao, ACE) are used to measure diversity within species, and beta-diversity metrics are employed using the Bray‒Curtis or binary Jaccard distance matrix to measure the differential abundance among species based on antibiotic exposure, probiotic consumption, delivery mode and feeding patterns. (2) Then, principal coordinate analysis (PCoA), nonmetric multidimensional scaling (NMDS), the unweighted pair-group method with arithmetic means (UPGMA) and heatmap of species clusters are used to conduct cluster and dimensionality reduction. (3) Furthermore, linear discriminant analysis (LDA) effect size (LEfSe), and Metastats methods are used to identify significant differences from the phylum to genus levels among groups. (4) Multivariable analysis is used to adjust for confounding factors and analyse the associations between clinical and microbiota data and identify the influencing factors of abundance and diversity of the mother-infant microbiota. (5) For longitudinal data analysis of gut microbiota changes over the first two years, a linear mixed effects model or multilevel mixed effects model are used. (6) A Dirichlet multinomial mixtures (DMM) model [40] is used for gut microbiota community type determination.

For *q*PCR data, parametric repeated tests such as ANOVA determine different groups over time, and nonparametric tests after transformation to log10 such as t tests are used for analysis.

All analyses are performed using Stata v.13.1 (StataCorp, College Station, TX), and visualization is performed using R statistical software v3.6.1 (http://www.r-project.org/) with the ggplot2 package. Statistical significance is evaluated using a 95% confidence interval and a two-tailed *p value* of < 0.05.

## Discussion

### Strengths and limitations of this study

There are several strengths in our study. First, the study is conducted in underdeveloped districts containing rural, suburban, and urban areas in Northwest China, which could be representative and provide evidence for a similar study in other less-developed districts worldwide. Second, not only exposure data during pregnancy, but also delivery and early life data of health outcomes are collected. Third, to ensure a high follow-up response rate, a detailed investigation plan had been formulated. Furthermore, for antibiotic and probiotic assessment, a questionnaire of knowledge and behaviour related to antibiotics for parents is conducted, and the Bayley Scales of Infant and Toddler Development (Bayley-IV) are used, to make the evaluation of the results more multidimensional. However, the study has some limitations. The registration of the study is relatively late due to the COVID-19 pandemic, but the normality of the cohort is not affected by late registrations. A possible selection bias exists in the study population because investigators recruited individuals who cooperated rather than a random sample, and the rate of antibiotic use varies during pregnancy, so a large sample size is needed. Moreover, due to limitations in medical knowledge and women’s migration for work, many participants will fail to complete the follow-up of the two-year study. However, we will keep close contact with the participants to limit loss to follow-up. In addition to the time points aligned with scheduled immunization and health services, it seems urgent that we combine the information with health service systems of different institutions for the integrity of the data. In the future, if the study budget and time permit, the follow-up time will be extended.

### Implications

The PBCC provides a multidimensional platform for studying antibiotic resistance and health outcomes during early life through a longitudinal perspective with a focus on the alteration of the gut ecology. The rich dataset from three representative areas will inform antibiotic and probiotic use for pregnant women and infants and contribute to establishing rational strategies on the use of antibiotics and probiotics for paediatricians, health practitioners, and drug administration policy-makers. In the study, we will extend the analysis of biological samples to breast milk, vaginal swabs and oral swabs from the same cohort in further studies. Furthermore, we are considering collaborating with microbiologists to create a biobank of probiotic strains to serve as stable selection samples for human use. Finally, we intend to combine data from different institutions (including primary health care units and the Centre for Disease Control) and multiple biological samples (including breastmilk, vaginal and oral samples) to explore new evidence between antibiotic/probiotic exposure during the perinatal period and health outcomes in early life.

## Data Availability

Data sharing is not applicable to this article as no datasets were generated or analysed during the current study.

## Abbreviations

AAD: Antibiotic-Associated Diarrhoea
ANC: Antenatal Care
ANOVA: Analysis of Variance
ARGs: Antibiotic Resistance Genes
BMI: Body Mass Index
ChiCTR: Chinese Clinical Trial Registry
DMM: Dirichlet Multinomial Mixtures
FAO: Food and Agriculture Organization
HIS: Hospital Information System
LEfSe: Linear Discriminant Analysis (LDA) Effect Size
PBCC: Pregnancy and Birth Cohort in north-western China
NMDS: Non-metric multidimensional scaling
PCoA: Principle Coordinate Analysis
PCR: Polymerase Chain Reaction
qPCR: Quantitative Polymerase Chain Reaction
REDCap: Research Electronic Data Capture
RR: Risk Ratio
SD: Standard Deviation
UPGMA: Unweighted Pair-Group Method with Arithmetic means
WHO: World Health Organization
